# Is thinking really aversive? A commentary on Wilson et al.'s “Just think: the challenges of the disengaged mind”

**DOI:** 10.3389/fpsyg.2014.01427

**Published:** 2014-12-09

**Authors:** Kieran C. R. Fox, Evan Thompson, Jessica R. Andrews-Hanna, Kalina Christoff

**Affiliations:** ^1^Cognitive Neuroscience of Thought Laboratory, Department of Psychology, University of British ColumbiaVancouver, BC, Canada; ^2^Department of Philosophy, University of British ColumbiaVancouver, BC, Canada; ^3^Institute of Cognitive Science, University of Colorado BoulderBoulder, CO, USA; ^4^Brain Research Centre, University of British ColumbiaVancouver, BC, Canada

**Keywords:** thinking, spontaneous thought, mind wandering, affect, enjoyment, consciousness, self-report

Spontaneous thought, often colloquially referred to as “daydreaming” or “mind-wandering,” is increasingly being investigated by scientists (for recent reviews, see Christoff, 2012; Andrews-Hanna et al., 2014; Smallwood and Schooler, 2014). In a recent article published in *Science*, Wilson et al. (2014) argue in support of the view (e.g., Killingsworth and Gilbert, 2010) that such thinking is predominantly unpleasant, and even emotionally aversive. While we were impressed with the enormous wealth of data collected by Wilson et al. and by the number of experimental manipulations carried out, we found their interpretations surprising in light of prior research. We applaud Wilson et al.'s detailed effort to investigate the content and affective qualities of “just thinking”—but upon examining their dataset, we find little support for their claims.

Wilson et al. make three central claims, as summarized in their article's abstract: (i) “participants typically did not enjoy spending 6–15 min in a room by themselves with nothing to do but think”; (ii) participants “enjoyed doing mundane external activities much more” than “just thinking”; and (iii) “many [participants] preferred to administer electric shocks to themselves instead of being left alone with their thoughts.” These claims were surprising to us because they contradict the findings from a substantial body of research on the affective qualities of thinking and daydreaming (Singer and McCraven, 1961; Killingsworth and Gilbert, 2010; Stawarczyk et al., 2011, 2013; Song and Wang, 2012; Andrews-Hanna et al., 2013; Diaz et al., 2013; Ruby et al., 2013; Tusche et al., 2014; the results from these studies are summarized in Table S1 in our Supplementary Materials).

After closely examining Wilson et al.'s data, we found very little support for their first and third central claims—similar to other independent, critical examinations of their dataset (e.g., Jabr, 2014; Nelson, 2014). We did find their second claim to be supported by their data—but for external activities that were engaging, and tailored to participants' personal interests, rather than “mundane.” Overall, we argue that it is impossible to draw meaningful conclusions regarding the “typical” affective qualities of spontaneous thought, given their enormous variability both within and across individuals (for a similar argument, see Gelman, 2014).

## Is “just thinking” enjoyable?

**Figure 1:**
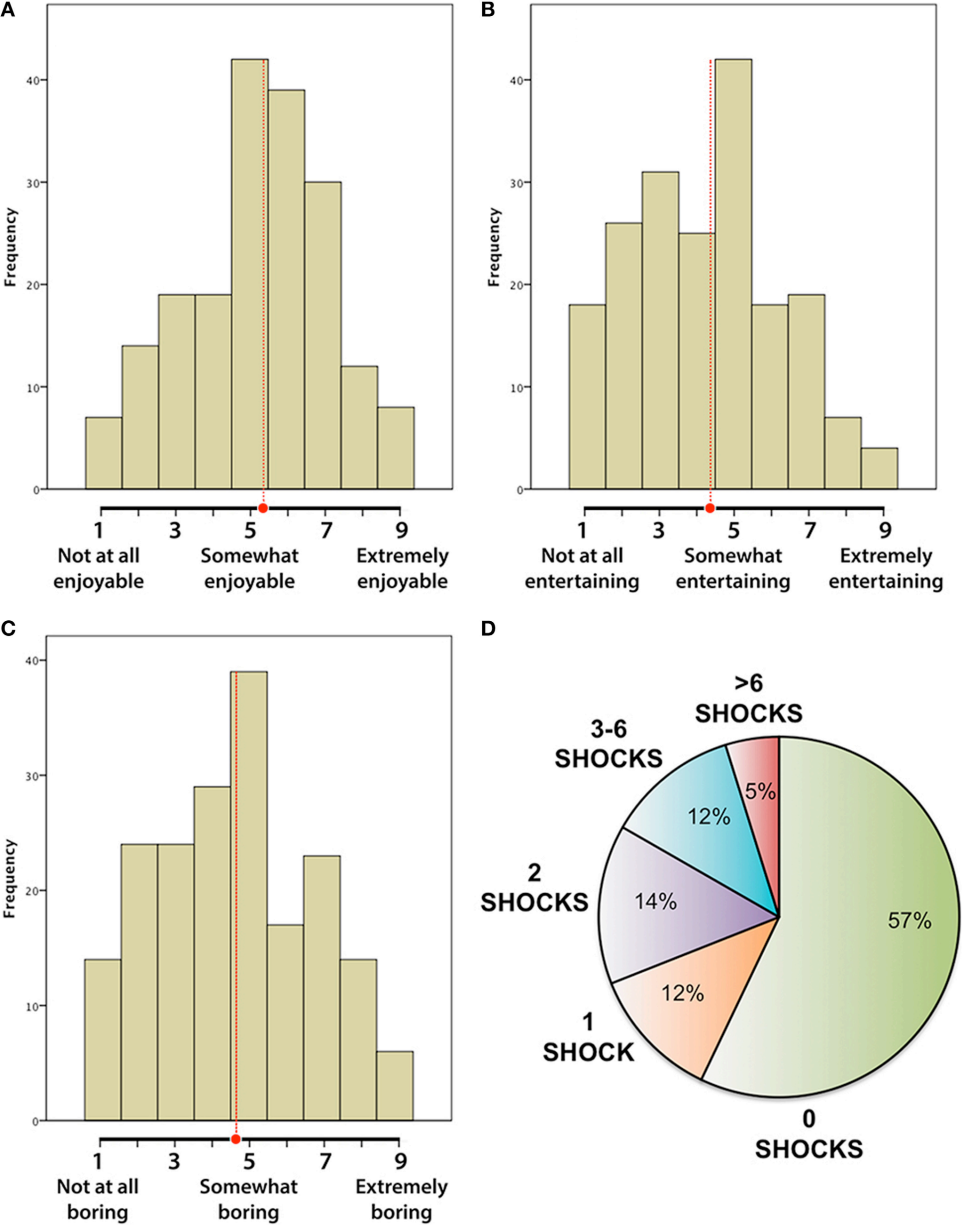
**First-person reports about the experience of just thinking from Wilson et al. Histograms (A–C) showing the distribution of self-reported enjoyment (A), entertainment (B), and boredom (C), during “just thinking” across 190 participants in Studies 1–7**. The mean scores across participants, indicated with red dots and dashed red lines, were **(A)** 5.21 (*SD* = 1.95), **(B)** 4.24 (*SD* = 2.04), and **(C)** 4.60 (*SD* = 2.14), respectively. These data clearly show that just thinking was somewhat enjoyable, somewhat entertaining, and somewhat boring—directly contradicting Wilson et al.'s claim that it was not enjoyable, and even aversive. **(D)** Number of shocks self-administered during a 15 min “just thinking” session in Study 10. Data are from the 42 participants who had previously said that they would pay money to not receive the shock. The data clearly show that a majority of participants prefer “just thinking” to receiving an electric shock.

In support of their first central claim, Wilson et al. present results from their Studies 1–7, reporting that “participants did not enjoy the experience [of ‘just thinking’] very much,” rating it overall as 4.94 on a 9-point scale of “enjoyability” (a composite of three scales; see their Table 1). However, as becomes clear upon consulting their original data (available at https://osf.io/cgwdy/files), the midpoints of the three component scales were “somewhat enjoyable,” “somewhat entertaining,” and “somewhat boring,” respectively. Figure 1 presents the distributions of self-reported enjoyment **(A)**, entertainment **(B)**, and boredom **(C)** associated with “just thinking” (Studies 1–7) from the authors' original dataset. Contrary to what Wilson et al. conclude, participants typically (i.e., on average) found just thinking to be somewhat enjoyable, somewhat entertaining, and somewhat boring. The distributions presented in Figure 1, however, also underscore the large variability in self-reported affect—variability that should caution us against drawing inferences about a single “typical” value.

## Are external activities preferable to “just thinking”?

In support of their second central claim that “mundane” external activities are preferable to “just thinking,” Wilson et al. present findings from their Study 8. Here, participants who engaged in external activities reported significantly higher enjoyment than participants who simply entertained themselves with their own thoughts. These results do show that participants enjoyed external activities more than “just thinking,” but these external activities were not banal or boring, as Wilson et al. seem to suggest by using the word “mundane” to describe them; they included “watching a television show or movie,” “playing a videogame,” “reading an enjoyable book or magazine,” and “looking at web pages (e.g., Facebook, YouTube).” Moreover, these activities were tailored to the personal interests of the participants, who themselves chose from an extensive list those activities they thought would be most entertaining. Finally, participants were free to switch between activities during the session.

Wilson et al. use the finding of higher ratings for personally-selected external activities to support the claim that “just thinking” is aversive, but such an inference is a *non-sequitur*, analogous to claiming that people find chocolate “aversive” and “not very enjoyable” simply because they consistently rate sexual activity as more enjoyable, more entertaining, and less boring. There is a large psychological distance between “not as enjoyable as watching TV” and “aversive.”

Wilson et al. also report that some participants disobeyed the “just think” instructions and “cheated” by engaging in external activities (e.g., checking cell phones). Wilson et al. consider this as evidence that just thinking is not enjoyable—but the inability, or unwillingness, to engage in a particular activity continuously for 12–15 min without a break or change of pace cannot be used to answer questions about the activity's inherent enjoyability in as straightforward a manner as Wilson et al. suggest. To return to the chocolate analogy, imagine a study in which subjects were made to sit in a room and eat chocolate without interruption for 12 min. Boredom, disgust, “cheating,” and messier problems would likely ensue. Could we then conclude that chocolate is “aversive” or “not very enjoyable”? There are innumerable activities that could be widely agreed to be enjoyable—but many would no longer be so, if engaged in continuously and in response to instructions rather than spontaneously.

## Would people rather administer electric shocks to themselves than “just think”?

In support of their third central claim that participants preferred shocking themselves to “just thinking,” the authors present the results from Study 10. In part 1 of this study, participants rated the pleasantness of several stimuli, including an electric shock. In part 2 of the study, participants were instructed to entertain themselves with their thoughts during a 15 min session; they were also told that if they wanted, they could receive the electric shock again by pressing a button. Most participants (57%) never shocked themselves at all, and only a small minority (17%) self-administered more than two shocks during the entire session (Figure 1D; see also our Supplementary Materials). Considering the length of the thinking session (15 min) and the brevity of the shock (presumably no longer than a second or two), the results from Study 10 show that nearly all participants preferred to spend the vast majority of their time “just thinking” rather than self-administering shocks.

What motivated a minority of participants to shock themselves during the “just think” period? Wilson et al. suggest that this behavior was motivated by a desire to avoid the aversiveness of “just thinking.” They conclude: “what is striking is that simply being alone with their own thoughts for 15 min was apparently so aversive that it drove many participants to self-administer an electric shock.” However, many findings from their dataset argue against this conclusion. First, participants in the shock study (including those who chose to self-administer shocks) did not describe “just thinking” as aversive, but rather as on average “somewhat enjoyable” (as in the first 9 studies). Second, the electrical shock was rated on average as only slightly unpleasant, even by those who said they would pay a small hypothetical sum of money to avoid another shock (*M* = 4.12, on a 9-point scale from “very unpleasant” to “very pleasant,” with 5 being neutral). Third, Wilson et al. found no statistical difference in enjoyment of “just thinking” between those people who shocked themselves and those who didn't (see p. 10 of their Supplementary Materials). Fourth, Wilson et al. collected open-ended first-person reports following the shock study asking subjects about their motivation for self-administering shocks, and the content of their thoughts during these “just think” sessions (these reports are reproduced in full in our Supplementary Materials). The data from participants' responses to these open-ended questions also belie Wilson et al.'s interpretations.

One of Wilson et al.'s open-ended questions asked, “Why did you choose or not choose to experience shock during the thinking period?” Of the 18 participants who shocked themselves, 14 state some form of curiosity about (or interest in) the quality of the shock or its effects as their motivation. Four of 18 subjects mention boredom as a reason, but given that boredom is not identical with unpleasantness, these reports do not provide support for Wilson et al.'s conclusion. Indeed, recall that Wilson et al.'s overall scale of “enjoyment” is a composite of three scales, including “boredom” (where higher reports of boredom resulted in a lower composite measure of “enjoyment”; see their Table 1). Reports of boredom therefore cannot be considered indicative of aversiveness or unpleasantness: participants consistently reported just thinking to be “somewhat enjoyable” and “somewhat entertaining” *in spite of* it being “somewhat boring.”

Another open-ended question from Wilson et al.'s dataset asked participants, “Please describe, in your own words, what you thought about during the Thinking Period.” Were participants' thoughts really so aversive that an electric shock was a welcome escape? Consulting the reports provides no support for this claim: subjects whiled away their time planning weekend parties, recalling sunny beach vacations, or anticipating the coming summer holidays. Of the 18 subjects who self-administered shocks, 12 reported explicitly positive, enjoyable thoughts about friends, beach getaways, etc.; five subjects reported predominantly neutral thoughts; and only one mentioned boredom. One subject reported high levels of distraction, but no subject reported negative or aversive thoughts.

## Wilson et al.'s findings (but not their interpretations) are consistent with prior research

Wilson et al.'s “just thinking” sessions largely overlap with the related forms of spontaneous thought known as daydreaming or mind-wandering: as Wilson et al. (2014) summarize the situation, “most subjects reported that… their mind wandered (89.0% responded at or above the midpoint of the scale), even though there was nothing competing for their attention” (p. 76). On a 1–9 scale from “not at all” (1) to “very much” (9), the average rating for the extent of mind-wandering in studies 1–7 was 6.94, suggesting that mind-wandering was extremely prevalent during the “just thinking” periods. Indeed, these scores appear to be some of the most extreme values Wilson et al. obtained on *any* of their self-report measures—considerably higher than the ratings of enjoyment, entertainment, boredom, and difficulty concentrating. Probably the safest conclusion to draw from Wilson et al.'s immense dataset about “just thinking” is that its defining characteristic, and dominant content, is none other than “mind-wandering.”

It is therefore important to examine how their results compare to prior research on the affective qualities of mind-wandering. Nine independent studies investigating the affective content of mind-wandering and related forms of self-generated thought contradict Wilson et al.'s conclusions, but not their actual data: across more than 4300 international participants and a variety of questionnaires, thought-sampling methods, and experimental settings (e.g., in the lab, at home, in daily life), mind-wandering is consistently reported to be on average mildly pleasant or mildly positive in nature (Singer and McCraven, 1961; Killingsworth and Gilbert, 2010; Stawarczyk et al., 2011, 2013; Song and Wang, 2012; Andrews-Hanna et al., 2013; Diaz et al., 2013; Ruby et al., 2013; Tusche et al., 2014; results summarized in Table S1). The results reported by Wilson et al. are fully consistent with these findings (Figures 1A,B), despite their own interpretations.

## Conclusions

Overall, Wilson et al.'s results provide little support for the conclusion that people find being alone with their own thoughts unpleasant or aversive. Instead, their results—like the results from many prior studies of the affective qualities of self-generated thought—show “just thinking” to be on average somewhat enjoyable. But behind these simple averages lies remarkable individual and situational variability. It is this variability that best characterizes the affective qualities of thinking, rather than any one static point along the continuum from aversiveness to enjoyment.

Spending time with our thoughts likely has complex and wide-ranging implications beyond momentary hedonic enjoyment. Spontaneous thoughts are perceived as revealing meaningful self-insight (Morewedge et al., 2014) and may play an important adaptive role in life-relevant problem solving (Baars, 2010) and providing meaning to our lives (Christoff et al., 2011). Even when it is less than enjoyable or entertaining, spending time with our own unstructured thoughts may increase our overall sense of well-being and life satisfaction. To paraphrase Pascal, the spontaneous mind may have its reasons, of which scientists still know rather little. Gaining a better understanding of these reasons remains a challenge to scientific research.

## Conflict of interest statement

The authors declare that the research was conducted in the absence of any commercial or financial relationships that could be construed as a potential conflict of interest.

## Supplementary material

The Supplementary Material for this article can be found online at: http://www.frontiersin.org/journal/10.3389/fpsyg.2014.01427/full
