# Supplementary material for: Is thinking really aversive? A commentary on Wilson et al.'s “Just think: the challenges of the disengaged mind”
Source: Front Psychol. 2014 Dec 9;5:1427. doi: 10.3389/fpsyg.2014.01427 (PMC4260464; doi:10.3389/fpsyg.2014.01427)
Supplement: Supplementary file 1 [file DataSheet1.DOCX]

**Is thinking really aversive? A commentary on Wilson et al.’s “Just think: The challenges of the disengaged mind”**

**Supplementary Online Materials**

**Kieran C. R. Fox^1^, Evan Thompson^2^, Jessica R. Andrews-Hanna^3^, and Kalina Christoff^1,4^***

^1^Department of Psychology, University of British Columbia, 2136 West Mall, Vancouver, B.C., V6T 1Z4, Canada

^2^Department of Philosophy, University of British Columbia, 1866 Main Mall, Vancouver, B.C., V6T 1Z1, Canada
^3^ Institute of Cognitive Science, University of Colorado Boulder, UCB 594, Boulder, CO, 80309-0594 U.S.A.
^4^Brain Research Centre, University of British Columbia, 2211 Wesbrook Mall, Vancouver, B.C., V6T 2B5 Canada

*** Correspondence:** Kalina Christoff, Department of Psychology, University of British Columbia, 2136 West Mall, Vancouver, B.C., V6T 1Z4, Canada.

kchristoff@psych.ubc.ca

**Keywords: thinking, spontaneous thought, mind wandering, affect, enjoyment, consciousness, self-report.**

**1. Prior studies of the affective qualities of self-generated thought**

A fairly substantial body of prior research has investigated questions and mental phenomena very similar to those addressed by Wilson et al. In the manuscript, we note that every one of these studies has found self-generated thought to be mildly pleasant or positive, on average, fully in line with Wilson et al.’s dataset (but contrary to their conclusions and interpretations). In this table we summarize the results of each of these studies.

*Table S1.* Summary of findings from prior studies of the affective qualities of self-generated thought.

| **Study** | **Sample size** | **Summary of emotional valence findings** |
| --- | --- | --- |
| ([Singer & McCraven, 1961](#_ENREF_6)) | 240 | “Most people reported that they enjoy daydreaming” [results as summarized in ([Singer, 1966](#_ENREF_5))] |
| ([Killingsworth & Gilbert, 2010](#_ENREF_3)) | 2250 | Of all ‘mind-wandering’ episodes, 42.5% were rated as ‘pleasant’; 31.0% as ‘neutral’; and only 26.5% as ‘unpleasant’ |
| ([Stawarczyk, Majerus, Maj, Van der Linden, & D'Argembeau, 2011](#_ENREF_9)) | 53 | On a scale of –3 (‘very negative’) to +3 (‘very positive’), the mean rating was 0.62 and 0.19, respectively, for the two experimental groups (i.e., mildly positive in both cases) |
| ([Song & Wang, 2012](#_ENREF_7)) | 165 | On a 1-5 scale (1 = ‘negative’; 3 = ‘neutral’; 5 = ‘positive’), mean rating was 3.32 (i.e., mildly positive) |
| ([Andrews-Hanna et al., 2013](#_ENREF_1)) | 76 | On a 0-10 scale (0 = ‘very negative’; 5 = neutral; 10 = ‘very positive’), average rating of spontaneous thoughts was 5.9 (i.e., mildly positive) |
| ([Diaz et al., 2013](#_ENREF_2)) | 1367 | Numerous questions asked on a 5-point (1-5) scale (1 = ‘completely disagree’; 5 = ‘completely agree’) “I felt happy”: mean = 3.16 “I enjoyed the session”: mean = 2.84 “I had negative feelings”: mean = 1.90 |
| ([Stawarczyk, Cassol, & D'Argembeau, 2013](#_ENREF_8)) | 67 | On a scale of –3 (‘very negative’) to +3 (‘very positive’), the mean rating for future-oriented spontaneous thoughts was 0.57 and for non-future-oriented thoughts, 0.46 (i.e., mildly positive in both cases) |
| ([Ruby, Smallwood, Engen, & Singer, 2013](#_ENREF_4)) | 85 | On a scale of 1 (‘not at all’) to 9 (‘completely’), the mean rating for the question “How positive were your thoughts?” was ~6.0, whereas the mean rating for the question “How negative were your thoughts?” was ~3.4.  Thoughts were significantly more positive than negative. |
| ([Tusche, Smallwood, Bernhardt, & Singer, 2014](#_ENREF_10)) | 30 | On a scale of -3 (negative valence) to +3 (positive valence), the mean ratings was 0.77. |

**2. Further details regarding our analysis of the results of the shock study (Study 10)**

In our commentary, and Fig. 1D, we report that 57% of participants refused to shock themselves even once during the ‘just think’ session of Study 10. We based this value on the absolute number of participants who did or did not shock themselves. Of 42 participants involved in this part of Study 10, there were 18 men and 24 women. 12 of the men, but only 6 of the women, chose to shock themselves at least once (18/42 = 42.9% who did shock themselves, and therefore 57.1% who chose not to). Because the sample was skewed toward including more women, Wilson et al. prefer to deal with proportional values (per gender) in their results, but this method does not change our conclusions at all. Using proportions only and controlling for gender, 67% of men (12 of 18) and only 25% of women (6 of 24) shocked themselves, again showing that a majority of participants (54% in this case) chose not to shock themselves whatsoever during the ‘just think’ session.

**3. Reproduction of the answers to the open-ended questions about first-person experience and motivation in the shock study (Study 10)**

The following reports were extracted from Wilson et al.’s own supplementary dataset, which we downloaded from <https://osf.io/cgwdy/files>.

Q1. Why did you choose or not choose to experience shock during the thinking period?

(Results tallied up by number of shocks they self-administered)

**0 shocks (N=24)**1. Because I felt that the unpleasant feeling might stray me away from the pleasant thoughts I was having.
2. I found the shock unpleasant before and did not want to experience it again. I also believed that the shock would disrupt my positive train of thought.
3. I didn't feel like experiencing an unpleasant shock.
4. It was quite unpleasant
5. Because I didn't like the shock and I wasn't required to shock myself so I didn't
6. I chose not to because I did not find it pleasant to begin with, so I did not want to put myself through it another time.
7. I did not enjoy it the first time
8. It was unpleasant the first time and I didnt want it to interfere with the postive thinking process that was required of me.
9. Since the goal of the thinking period is to entertain myself, experiencing shock won't get myself entertained.
10. I chose not to experience shock because I didn't like it
11. It was unpleasant.
12. I just didn't care to use them, I was content without them.
13. The shocks were unpleasant, didn't want to feel it again.
14. I didn’t want them. I was content with my thoughts and didn’t need the shocks
15. Because it was unpleasant
16. I didn't need to feel the discomfort for any reason.
17. That was not very related to thinking of pleasant experience.
18. I did not see any point in shocking myself. I did not need that to entertain myself.
19. The shock is unpleasant
20. I told myself that I would only deliver a shock if I got really bored, so I kept myself from getting bored. My thoughts were much more appealing than shocking myself. After the first few minutes, I didn't even think about how I could shock myself anymore.
21. I wasn't sure if I wanted to or not. I was getting really bored by the end and was about to click it just for fun but the session ended.
22. It would have been unpleasant and there was no reason to do so
23. The shock was not particularly enjoyable and I preferred to not experience it again
24. I did NOT choose to experience the shock again because it hurt. I would never hurt myself knowingly! I like to be happy and feel good all the time. No pain, life's a game! :P

**1 shock (N=5)**1. To see if I still felt the same way about it
2. I was bored. I just wanted to feel it again.
3. I was starting to get drowsy and was wondering if the shock would make me more alert.
4. I wanted to see what would happen if I did shock myself. Would the study end, or keep going? Also, I wanted to see if the shock felt as painful the second time as it did the first time
5. I was curious about how my thoughts would change if I took the shock

**2 shocks (N=6)**1. Curiosity
2. I was bored and wanted to see what it felt like to be shocked again. The feeling in my foot was interesting.
3. I am not really sure why I chose to experience shock during the thinking period, other than the fact that I may have been a bit bored and wanted to try the shock once more.
4. Because I know that I'll be asking about experiencing shock so I did it.
5. I chose to willingly shock myself because I was so bored during the thinking period that I chose to experience the mild unpleasant shock over the oppressive boredom. Receiving the shock made me feel like I was being mildly stimulated, something I'd prefer over no stimulation. Plus, the sensation from the shock was slightly refreshing for thinking.
6. The first time I chose to receive the shock was to recall what it felt like. It was so fast the first time, and I had been anticipating a very unpleasant shock, but this one wasn't bad at all. The second time I chose to receive the shock was because I wasn't particularly sure if I could only get it ONCE within the 10-20 minute Thinking Period. For all I knew, there was only one shock per thinking period.

**4 shocks (N=3)**1. I chose to experience shock because I wanted to experience the interesting sensation I felt earlier in the experiment just to remind myself of what it felt like, and then after doing it once, I sort of liked the shock and proceeded to shock myself a few more times.
2. I thought that it felt cool and I wanted to listen to where the sound of the shock was coming from.
3. To remember what it felt like. Also, I seemed to habituate to the stimuli by around the 3rd shock.

**5 shocks (N=1)**1. I was curious to see what the shock was like again, and then I was wondering if I kept the shock button pressed down, if it would deliver a continuous shock, but it took me a few times to decide to keep the button pressed down-- I quickly realized that it only delivers the one small and brief shock, not a continuous one.

**6 shocks (N=1)**1. I mainly used it to wake myself up and I found the feeling of it very interesting. I thought it would hurt, but it did not feel bad.

**9 shocks (N=1)**1. I was bored, didn't hurt=, so why not

**190 shocks (N=1)** **(this subject thought that he administered 60 shocks)**
1. I was interested in the amount of pain that it would cause, to find that it didn't really bring on any.

Q2. Please describe, in your own words, what you thought about during the Thinking Period.

(Results tallied up by number of shocks they self-administered)

**0 shocks (N=24)**1. I thought it was a nice time to relax and let my mind wander a bit. I noticed that I was focusing a bit more on the three topics rather than anything pleasant that came to my mind, but it was still nice and peaceful.

2. Vacationing with my family, driving my car, living in a new place, winning a race, running track, visiting my old elementary school, having a romantic relationship or partner, and the devices, motives, and lives of the experimenters outside the room.

3. At first I thought about the objects in the room and the sounds from outside. I thought about why I was given the shock as my option and if people ever chose it. At one point I realized I was listening to music in my mind, and then I decided to continue with it because it was pleasant. My mind flicked back and forth to different images and scenes where I was doing something pleasant, like visiting a place I've never seen before or making food. I also imagined a nice breeze because it's hot in this room.

4. I had a headache so I thought a lot about being back in my room and going to sleep. I thought a lot about my boyfriend. I thought about upcoming stressful events, and things that needed to be done even though I tried not to. I was quite bored, so I kept wondering how much time was left and hoping that it would end soon. I fought sleep and probably thought most about being back in my bed.

5. At first I thought about family and friends then started thinking about different songs. Probably because I was listening to music on my way here

6. I thought about my summer and what my plans are for it. I also thought about the rest of my semester rather than thinking about finals being almost over. I almost forgot to think about my apartments for next year, but I thought about it some. Mostly random thoughts came into my head such as Foxfield Races, my sorority, and how I am going to move all my stuff out of my dorm at the end of the semester.

7. Winning and enjoying the relief of finally reaching my goals. Getting over past emotional troubles and moving on to better things. Realizing that the year is almost over and that I won't be living with my roommate again next year who has grown to be one of the best friends I have ever had.

8. I thought about doing well on the LSAT for law school. I thought about how tired I was and wanted to take a short nap. I was also nervous for a good amount of time that I will be surprisingly shocked despite not being asked for.

9. I am thinking about the dance party that I am going to this coming Saturday and the conversations that I have had before with my friends and my family. Also, I think about the upcoming summer vacation that I am going to go back to my home country and staywith my parents again. Moreover, something about the romantic moments jumped up into my mind during the thinking period.

10. I thought about painting and what I was going to paint later. I thought about dancing and singing and about what I was going to do this weekend. I thought about what was in the room and my surrounding environment. Then I started moving around my cursor beause I got pretty bored and then started to think about what I want to eat for dinner. Then I was just sitting there thinking nothing for a while and then wondered what time it was and then the timer went off.

11. A lot of different things, from what I would be doing later on, to the pictures and sounds I experienced earlier, to an upcoming quiz I have in my 12:30 class to song lyrics and nothingness.

12. I thought about wandering around a really lush green forest. It was raining and the mud was really bright red. The rain was making noise and it was humid.

13. Mainly just thought about music. Played songs in my head, songs I liked. Outside of that, I didn't think too much about anything else.

14. Things that had happened during the day

If I pushed the button to get the shock would i get our early

things i had bought at the store a few days ago

my future house

15. I thought about my past enjoyable events and my family.

16. I first thought about my dogs and how seeing them meant I was going home which is a very happy thought. Then I thought about how it is different coming home from college while everyone else is still in school. Then I relived my lacrosse years in high schol and all the wins I contributed to. Then I thought about how I was as a leader on the team and how i'm a leader within organizations I'm a part of and how I could use those qualities more in my relationships with people, but also how I like dealing with eople and how interactions with friends can be awesome.

17
1. I thought about going to some Chinese rural area for a break during the coming summer.
2. some traditional Chinese food that I could have after when I come back home.
3. my new hairstyle, the options I have
4. what to have for dinner tonight, probaby pav.
5. I imaged attending my friend's wedding and there were a lot of interesting things going on. Everyone was so happy and beautiful, the scene was nothing but perfect. We took a lot of photos together on the beach.
6. the lamp in the room is the sme as the one I have in my apartment.
7. I was trying to figure out how the shock thing works, how it is controlled by the no. 5 key, and what modulates the strength of the shock.

18. I thought about going to my brother's graduation, going to the beach this summer, going to the Dispatch concert on my birthday. I also thought about Fox Fields, my sorority family, and what its going to be like living in my apartment next year.

19. I spent most of the time thinking about finishing out the semester, my grades, and lining up a job for the summer.

20. I first began to think about my summer plans. I thought about how nice it will be to relax on the beach with my best friends from high school in just a few weeks. And then I thought about how excited I am to work at a beautiful camp in North Carolina. I pictured myself as a lifeguard with all of the campers around. I thought about throwing a summer pool party, and making the food and flower arrangements with my mom, which are both things we love to do. And then when I began to get tired, I started thinking about how nice it will be after exams are over to fall asleep without having to set an alarm for the morning. I looked back on my favorite memories from this year on Grounds, and then the bell rang.

21. It was hard to think about anything in particular. My mind was pretty blank but I was trying to bring pleasant thoughts into my head but for some reason it was hard to bring anything to mind. I'd say I mostly thought about all of the things that I have o do today and for the rest of the week, but I was trying to push those out and just think of pleasant things...I think this is what caused my mind to be slightly blank.

22. It tried to think about things I was looking forward to this summer, like my trip to Boston and my summer job which is at a Kindergarten. I had Mirrors by Justin Timberlake stuck in my head though, so about 40% of the time I was singing that in my head or trying not to think about it. I also wondered about the new store Bluetique that opened on the corner and what kind of stuff they might have. For a small period of time I thought about how the show The Office would end, mostly because I really want Michal to make a guest appearance but I don't know how they would make that happen.

23. First, I was thinking about the Thinking Period itself, my awareness of my perception of how time was passing, and wondering how this factored into the study -> Then, I thought about riding bikes a bit and realized that it was a silly answer to the earlier question because what I really wanted to do was... -> Make a story in my head, doing a few scenes over-and-over again until they were exactly right, but then... -> My thoughts wandered to stories I had read previously and scenes in them -> "What was hat character like?"; "Who was the author of that novel?"; "It was not technically very good, was it?"; -> Then back to my story and back and forth a few times -> "Oh no, am I dozing off a bit?" -> "Probably not, I'm just relaxed."

And then it was over

24. I thought about where I would go on my trip to Europe and all the thing I would see. I imagined just relaxing in a park somewhere where it is nice and quiet. I thought about having fun with my friends on my birthday. Mostly I thought about what I would do on Graduation day. How I would see my family and friends all together and enjoy the ceremony. As I let my thoughts wonder I figured out what kind of cake I want to make and how I want to decorate it. Orange and blue icing! Go HOOS! Because of that, this tinking period was very productive. Thank you! :D

**1 shock (N=5)**
1. During the Thinking Period I thought about my previous weekend and the events that occurred on Saturday at the Gala. I also thought a little about next weekend and what I would do since it is Spring Fling.

2. I'm going to Turks and Caicos in the summer, so I thought a lot about being on the beach with my family and friends there. I thought a little bit about volleyball, since I just came back from nationals in Dallas with the Men's Club Team. I was remembering that trip. Mostly I was thinking about date functions this weekend and imagining what they would be like.

3. This is the first time today I have been able to sit and think, so it was relaxing. I thought about vacations I would want to take, and dream scenarios for this summer.

4. I thought about going home, and what I was going to do. I thought about foods that my mom makes that I like, activities at home i like doing, and pets. I thought about family members that I havent seen in a while, and friends that i havent seen recently. thought about the swim practice i had this morning, and how i am tired from it. I thought about swimming at home, and how it will be different from my training here at UVA.

5. I enjoyed the thinking period. It gave me time to think about life without distractions or worries

**2 shocks (N=6)**
1. I thought about summer, and all of the things I enjoy doing during the summer. Specifically, I thought a lot about spending time at the cabin and on the lake. I also spent a little bit of time thinking about running, but not too much time. My thoughts also wandered to plans that I had later tonight.

2. I mainly just had songs stuck in my head. I thought about a few of my favorite bands as well as past concerts I had attended. I then thought of the beach and of summer as well as spending time outside.

3. I thought the thinking period was enjoyable in that it allowed me to ponder over certain things alone, without any distractions or other obligations in the way. It was a good way to regulate how my thoughts entered my mind and stayed fresh in them. In addition, the thinking period pushed for trying to make the experience enjoyable, so I was really only thinking happy thoughts and overall was content with my situation.

4. I thought about many stories ( fictional and non fictional ) that I've read, watched, experienced in real life. I thought about sport I play, game I play, song I used to sing. fortunately, the lyric that I'm trying so hard to remember just pops up right aay. it's really a good thing

5. I was incredibly bored. The only mild stimulation I received was from observing my surroundings and commenting on them in my mind and from the mild electrical shocks.

6. I thought about what I would say if I won an Academy Award for a good portion of the time. I also thought about what I would say on a talk show with Ellen DeGeneres or Oprah -- this would, of course, be in an ideal world. So these thoughts were very hypthetical, not really based on personal experience. A few times, however, I was thinking about my surroundings (the number pad for the electric shock, the computer's buzz, etc). I kept wondering if there was a camera watching me, just as I've seen in vidos of psyche studies.

**4 shocks** (N=3)
1. I thought a lot about the end of finals week and about going home for summer. I thought about the nostalgic feeling there is at the end of a semester and it made me feel happy inside. I thought a lot about making plans for summer with friends and where could travel to and things that I could do for fun.

2. I mainly thought about movies. In particular, I thought about the super hero movies (The Avengers being a main one), which I realized how nerdy that was. However, that did not stop me from thinking about what it would be like to have super powers. Since te electric shock was my stimuli choice, I thought about how cool it would be to have electric powers. Then my mind went on to think about the new Man of Steel movie coming out this summer. Thinking about Superman led me to thoughts about the classic themesong from the movies. This is when my mind began to wander. I began to observe the room briefly. If this room had been a prison, I imagined how I would try to escape. I would probably try the Shawshank Redemption method and make a hole in the wall behind ne of these sound things on the wall. However, then I noticed the camera in the room and realized that my plan would never work. Then I decided to shock myself again because I thought it felt cool and I wanted to listen to where the shock was coming from.

3. I thought about what may happen over the summer, a few memorable experiences with my friends, and the fun that may take place over this next weekend. Along with this were a few random thoughts.

**5 shocks (N=1)**At first I tried to focus on the positive and pleasant upcoming events that I listened, but I found myself getting distracted by the smallest sound from outside the room. I also was interested in the shock, and hesitated at first, then pressed the shock btton probably 5 times-- I was trying to figure out if the shock would continue if I kept the "5" pressed down, but took me a while to decide to actually try it out. The rest of the time, I found my mind wandering and thinking about my plans for tonight, gtting through finals, and for a while, I felt like I was thinking about nothing really.

**6 shocks (N=1)**1. I thought about the time that my family and I went to the beach. My friend also came with me and we had a great time. We went out in the ocean and got clobbered by waves. My friend, my brother, and I also went kayaking in the sound that was very close to he beach house we rented. It was really relaxing and we brought lunch to eat with us on the land by the sound.

**9 shocks (N=1)**1. I thought about what I would do when I leave the study, thought about what occurred over the weekend. Though about my schedule for tomorrow.

**190 shocks (N=1)**

1. I thought about what i could possibly think about. This started out with thinking of ways to possibly reinvent the keyboard, which transitioned to me playing with the shock. I tried clicking it as fast as i could, then i thought about how the current wa flowing through the wires and into my ankle. After getting bored with that, I began thinking of making music with the noise the shock generator was producing. This then transitioned into my thinking of what do with my stock portfolio, and my investment strategy.

**4. References**

Andrews-Hanna, J. R., Kaiser, R. H., Turner, A. E. J., Reineberg, A. E., Godinez, D., Dimidjian, S., & Banich, M. T. (2013). A penny for your thoughts: dimensions of self-generated thought content and relationships with individual differences in emotional wellbeing. *Frontiers in Psychology, 4*. doi: 10.3389/fpsyg.2013.00900

Diaz, B. A., Van Der Sluis, S., Moens, S., Benjamins, J. S., Migliorati, F., Stoffers, D., . . . Van't Ent, D. (2013). The Amsterdam Resting-State Questionnaire reveals multiple phenotypes of resting-state cognition. *Frontiers in Human Neuroscience, 7*.

Killingsworth, M. A., & Gilbert, D. T. (2010). A wandering mind is an unhappy mind. *Science, 330*, 932.

Ruby, F. J., Smallwood, J., Engen, H., & Singer, T. (2013). How self-generated thought shapes mood—the relation between mind-wandering and mood depends on the socio-temporal content of thoughts. *PLoS ONE, 8*(10), e77554.

Singer, J. L. (1966). *Daydreaming: An introduction to the experimental study of inner experience*. New York: Crown Publishing Group/Random House.

Singer, J. L., & McCraven, V. (1961). Some characteristics of adult daydreaming. *The Journal of Psychology, 51*, 151-164.

Song, X., & Wang, X. (2012). Mind wandering in Chinese daily lives–an experience sampling study. *PLoS ONE, 7*(9), e44423.

Stawarczyk, D., Cassol, H., & D'Argembeau, A. (2013). Phenomenology of future-oriented mind-wandering episodes. *Frontiers in Psychology, 4*.

Stawarczyk, D., Majerus, S., Maj, M., Van der Linden, M., & D'Argembeau, A. (2011). Mind-wandering: phenomenology and function as assessed with a novel experience sampling method. *Acta Psychol (Amst), 136*(3), 370-381. doi: 10.1016/j.actpsy.2011.01.002

Tusche, A., Smallwood, J., Bernhardt, B. C., & Singer, T. (2014). Classifying the wandering mind: Revealing the affective content of thoughts during task-free rest periods. *Neuroimage*.
